# Assessment of Frequency and Predictive Value of Comorbidities in Patients With Disorders of Consciousness in the Acute Setting

**DOI:** 10.1089/neur.2023.0120

**Published:** 2024-03-14

**Authors:** Gennaro Saporito, Luca Gentili, Angelo Cacchio, Alfonsina Casalena, Stefano Necozione, Alessandro Ricci, Federica Venturoni, Franco Marinangeli, Francesca Pistoia

**Affiliations:** ^1^ Department of Biotechnological and Applied Clinical Sciences, Intensive Care, and Pain Treatment, University of L'Aquila, L'Aquila, Italy.; ^2^ Department of Anesthesia and Intensive Care Unit, Mazzini Hospital, Teramo, Italy.; ^3^ Department of Life, Health and Environmental Sciences, Intensive Care, and Pain Treatment, University of L'Aquila, L'Aquila, Italy.; ^4^ Neurology Unit, Mazzini Hospital, Teramo, Italy.; ^5^ Department of Neurosurgery, San Salvatore Hospital, L'Aquila, Italy.; ^6^ Department of Anesthesiology, Intensive Care, and Pain Treatment, University of L'Aquila, L'Aquila, Italy.; ^7^ Department of Neurology, San Salvatore Hospital, L'Aquila, Italy.

**Keywords:** coma, comorbidities, CoCos, minimally conscious state, vegetative state (unresponsive wakefulness syndrome)

## Abstract

Medical comorbidities are frequent in patients with disorders of consciousness (DoC) and their impact on outcomes is under investigation. The aim of this study was to investigate patients with DoC in the acute stage and the influence of comorbidities. Patients admitted to intensive care units and neurological units with a diagnosis of coma, vegetative state/unresponsive wakefulness syndrome (VS/UWS), and minimally conscious state (MCS) were investigated through the Glasgow Coma Scale (GCS), the Coma Recovery Scale – Revised (CRS-R) and the Comorbidities Coma Scale (CoCos). Forty-three patients (21 men and 22 women; mean age at admission: 60.4 ± 21.0) were included in the study. The most frequent diagnosis at admission was coma (72%) followed by VS/UWS (14%) and MCS (14%). The most frequent brain injury was subarachnoid hemorrhage (46%). At the 6-month follow-up, 19 patients had died (44%), 15 showed a full recovery of consciousness (35%), 7 were in a condition of emergence from MCS (16%), and 2 showed a persistent VS/UWS (5%). Forty-two (98%) patients showed at least one comorbidity: presence of life-support device (92.9%), anemia (76.2%), arterial hypertension (66,7%), hydrocephalus (45.3%), and respiratory infections (45.2%) were those most frequently reported. At the Multivariable Cox regression, the presence of renal disease (hazard ratio [HR] 33.37; *p* = 0.033) and malnutrition (HR 14.52; *p* = 0.001) were predictors of missed recovery of full consciousness. Although adverse outcomes are generally predicted by the severity of brain damage, the presence of medical comorbidities in an acute phase could influence outcomes and long-term prognosis.

## Introduction

Disorders of consciousness (DoC) include coma, unresponsive wakefulness syndrome (UWS) formerly known as the vegetative state (VS), and minimally conscious state (MCS)^1–4^ Coma results from a severe acquired brain injury with an admission Glasgow Coma Scale (GCS) of ≤8.^5^ Coma can evolve in different directions: some patients die after a few days or hours as a result of severe brain damage, whereas others survive the acute phase with the support of the intensive care unit (ICU). Among surviving patients, not all recover a normal state of consciousness. Some of them move to a condition of UWS/VS, with a possible further improvement toward a condition of MCS or emergence from MCS.

Previous evidence showed that the outcome of patients is influenced not only by the primitive brain damage but also by the presence of medical comorbidities.^6–8^Such comorbidities contribute to the instability of vital functions in the acute phase and decrease the recovery chances in the long term by interfering with the whole process of recovery.^9^ The prognostic impact of comorbidities on survival and functional improvement has been previously explored, albeit in the absence of a scale specifically developed to investigate the clinical profile of severely brain-injured patients. With this limitation, available data showed that comorbidities are common in these patients and that some of them influence recovery of consciousness and outcomes.^6^ In particular, we already showed that the severity of comorbidities and the presence of ischemic or organic heart diseases were the strongest predictors of death, together with increasing age.^6^ Moreover, respiratory diseases and arrhythmias without organic heart diseases were negative predictors of full recovery of consciousness and functional improvement.^6^ Other studies, mainly focusing on the frequency of comorbidities in DoC, reported pneumonia, pain, pressure ulcers, urinary tract infection, and limb spasticity as the most frequently recognized comorbidities.^10^ Clinical comorbidities have been reported to predict poor outcomes, especially when they are multiple and occur in combination, thus increasing the complexity of patients and their degree of clinical instability.^9^ We later developed and published a new scale, the Comorbidities Coma Scale (CoCoS), to be used for the assessment of medical comorbidities in these patients.^10^ The scale is now available and validated in English and Russian.^10,11^ This scale can add value to current research by favoring uniformity among studies, reproducibility of results, and data comparison.

The aim of the present study was to identify the impact of comorbidities on the outcomes of severely brain-injured patients with coma, UWS/VS, and MCS using the CoCoS in the acute setting.

## Methods

### Study population

Patients admitted to the Neurology and Stroke Unit and the Intensive Care Unit of the San Salvatore Hospital of L'Aquila and to the Intensive Care Unit of the Mazzini Hospital of Teramo within a 2-year period were screened for the inclusion in the study 1 week after admission. Inclusion criteria were a diagnosis of coma, UWS/VS or MCS after traumatic brain injury (TBI), stroke, anoxic encephalopathy (AE), or other acquired brain injuries, and the presence of signed informed consent by the proxy or surrogate to participate in this observational study. Diagnosis of coma, UWS/VS, or MCS were made through repeated observations during a 1-week period according to clinical criteria, and using the GCS and the Coma Recovery Scale – Revised (CRS-R).^1–5,12^ Included patients were assessed and followed to ascertain the presence and the burden of medical comorbidities using the CoCoS.^10^ The impact of comorbidities on 6-month outcomes was then evaluated. The study was approved by the Internal Review Board of the University of L'Aquila (n. 07/2020).

### Included participants

Overall, 43 patients (21 men and 22 women, mean age 60.4 ± 21.0) with a severe acquired brain injury and a GCS of ≤8 in the acute phase, in the first hours after admission to the ICU) were included in the study. Demographic and clinical characteristics of the included patients at baseline are reported in Table 1. The diagnoses at the time of inclusion were coma (*n* = 31; 72%) followed by VS/UWS (*n* = 6; 14%) and MCS (*n* = 6; 14%). The primitive brain damage, responsible for consciousness impairment, was subarachnoid hemorrhage (SAH) (*n* = 20; 46%) in most of the patients, followed by TBI (*n* = 8; 19%), intracerebral hemorrhage (ICH) (*n* = 6; 14%), ischemic stroke (*n* = 6; 14%), post-anoxic encephalopathy (*n* = 2; 5%), and metabolic encephalopathy (*n* = 1; 2%). The median GCS score at the inclusion in this study was 5 (minimum 3 maximum 14), the median CRS-R score was 5 (minimum 0 maximum 19) and the median CoCoS total score was 10 (minimum 0 maximum 20).

**Table 1.** Demographic and Clinical Characteristics of the Included Patients at Baseline

	n^a^	%	Mean age±SD
Gender			
Men	21	49%	53.4 ± 21.5
Woman	22	51%	67.1 ± 18.6
Diagnosis			
Coma	31	72%	62.2 ± 21.1
VS	6	14%	59.7 ± 24.4
MCS	6	14%	52.2 ± 18.5
Brain injury			
SAH	20	46%	60.1 ± 18.4
TBI	8	19%	56.1 ± 26.3
IS	6	14%	75.0 ± 27.5
ICH	6	14%	60.0 ± 11.1
ME	1	2%	52
PAE	2	5%	42.5 ± 27.6
Follow-up evaluation			
Caregivers	4	9%	
Trained specialists	39	91%	

^a^ Number of patients included in the study.

SD, standard deviation; VS, vegetative state; MCS, minimally conscious state; SAH, subarachnoid hemorrhage; TBI, traumatic brain injury; IS, ischemic stroke; ICH, intracerebral hemorrhage; ME, metabolic encephalopathy; PAE, post-anoxic encephalopathy.

### Measures

For all patients, the level of consciousness was evaluated through the GCS and the CRS-R.^1–5,12^ The GCS is composed of items about eye, verbal, and motor responses, with a total GCS score ranging from 3 (completely unresponsive patient) to 15 (responsive patient). The GCS can quantify the behavioral responsiveness of patients immediately after the brain injury but it does not allow qualitative discrimination among VS, MCS, and emergence from MCS. Conversely, the CRS-R is both a quantitative and a qualitative measure, able to capture consciousness transitions along the clinical spectrum of DoC following coma.^13^ The CRS-R includes scales for auditory function, visual function, motor function, oromotor/verbal function, communication, and arousal. The main difference with the GCS is that the CRS-R implies a qualitative assessment of key behaviors that can denote a transition to MCS or emergence from MCS to full consciousness; that is, the complete recovery of the awareness of self and surroundings, denoted by the ability to respond, even basically, to environmental stimuli. On the other hand, the condition “emergence from MCS” can be considered the step immediately preceding the recovery of full consciousness, being characterized by the reappearance of specific key behaviors (functional object use and functional communication), that are considered favorable prognostic elements for recovery.

As recommended, the CRS-R assessment was made by trained specialists, starting from 1 week after admission. During the first 4 weeks, when a greater variability may be expected, the clinical assessment was performed at least three times a week. Later, the clinical assessment was based on weekly evaluations. Fluctuations of the scores have been carefully discussed for each patient, and a consensus has been reached for meaningful changes denoting a real consciousness transition.

All comorbidities already present at the time of admission and those of new onset were recorded through the CoCoS.^10^ The CoCos includes 24 categories, with scoring being based on the presence/absence of specific comorbidities. Specifically, for each category, scoring is based on the presence/absence of specific comorbidities and their severity (score range 0–3). Adding up the individual scores gives a total score, which indicates the cumulative burden of comorbidities according to the following cutoff scores: 0: no comorbidities; 1–24: mild comorbidities; 25–48: moderate comorbidities; 49–72: severe comorbidities.

### Outcomes

All deaths occurring within the period of follow-up were recorded. Among surviving patients, the outcome was evaluated at 6 months by using the CRS-R. Deaths and outcomes after discharge from aforementioned units were identified by telephone interview.

The following outcomes were considered: (1) death; (2) full recovery of consciousness, identified as the complete recovery of self-awareness and awareness of surroundings, denoted by the ability to respond, even basically, to environmental stimuli; (3) unchanged consciousness as compared with the first assessment; and (4) state of emergence from MCS, characterized by the reappearance of specific key behaviors (functional object use and functional communication), which are considered favorable prognostic elements for later recovery.^4^

### Statistical analysis

A descriptive statistic of demographic and clinical variables of patients was performed. Data were presented as means (± standard deviation [SD]) and as medians (minimum [min]; maximum [max]). Group differences in demographic and clinical data were assessed using non-parametric tests (chi square [v2] and Kruskal–Wallis analysis). Correlation between changes in clinical scores, calculated as changes from initial values, and demographic and clinical numerical or ordinal variables were assessed by Spearman's rho coefficient. Long-term survival outcomes were presented in the form of a Kaplan–Meier survival curve across categories of diagnosis. Follow-ups were calculated from the date of the acute event to death or censoring. Survival durations were also compared across diagnosis categories using a log-rank test for trend. To assess the influence of comorbidities, we used a univariate Cox proportional hazards regression analysis. Among the independent variables, GCS and CRS-R were dichotomized as ≤5 versus >5 based on the median value of the distribution. Total CoCos score was dichotomized ≤10 versus >10 based on the median value. Individual comorbidities were dichotomized as 0 (absent) or 1 (present).

Multivariable Cox proportional hazard regression analysis were applied considering all the variables at univariate Cox regression with a *p* value <0.05. All data were analyzed with Jamovi software 2.3.24.^14^

## Results

### Clinical profile of patients and comorbidities

**FIG. 1.**
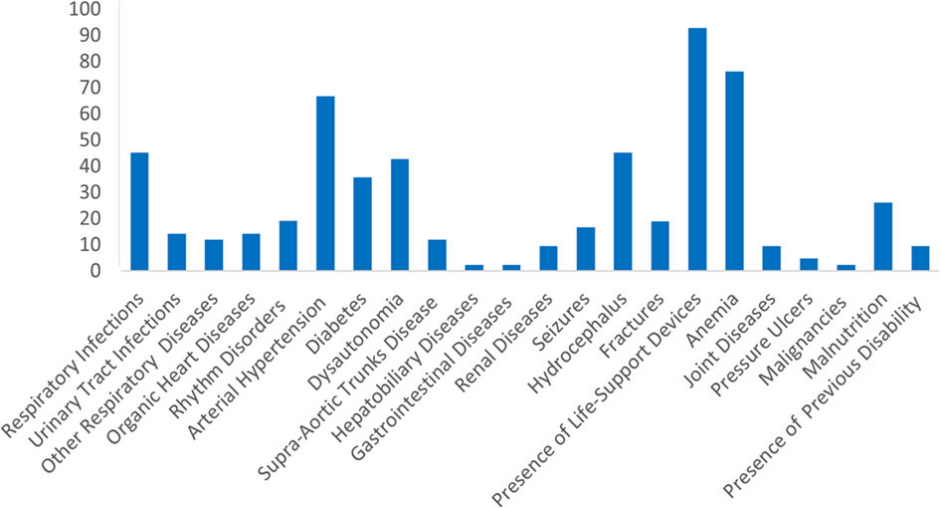
Frequency of comorbidities in the included patients.

**FIG. 2.**
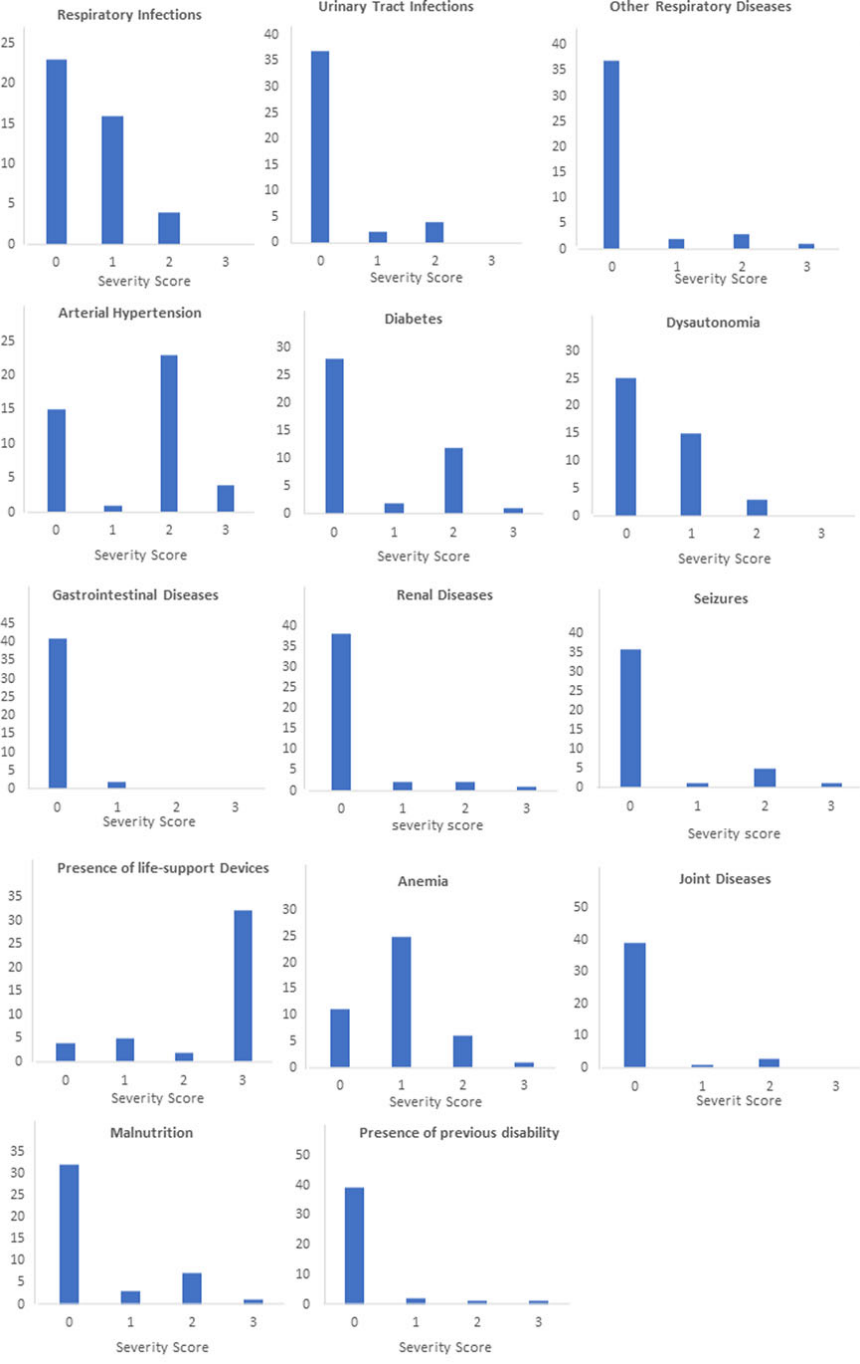

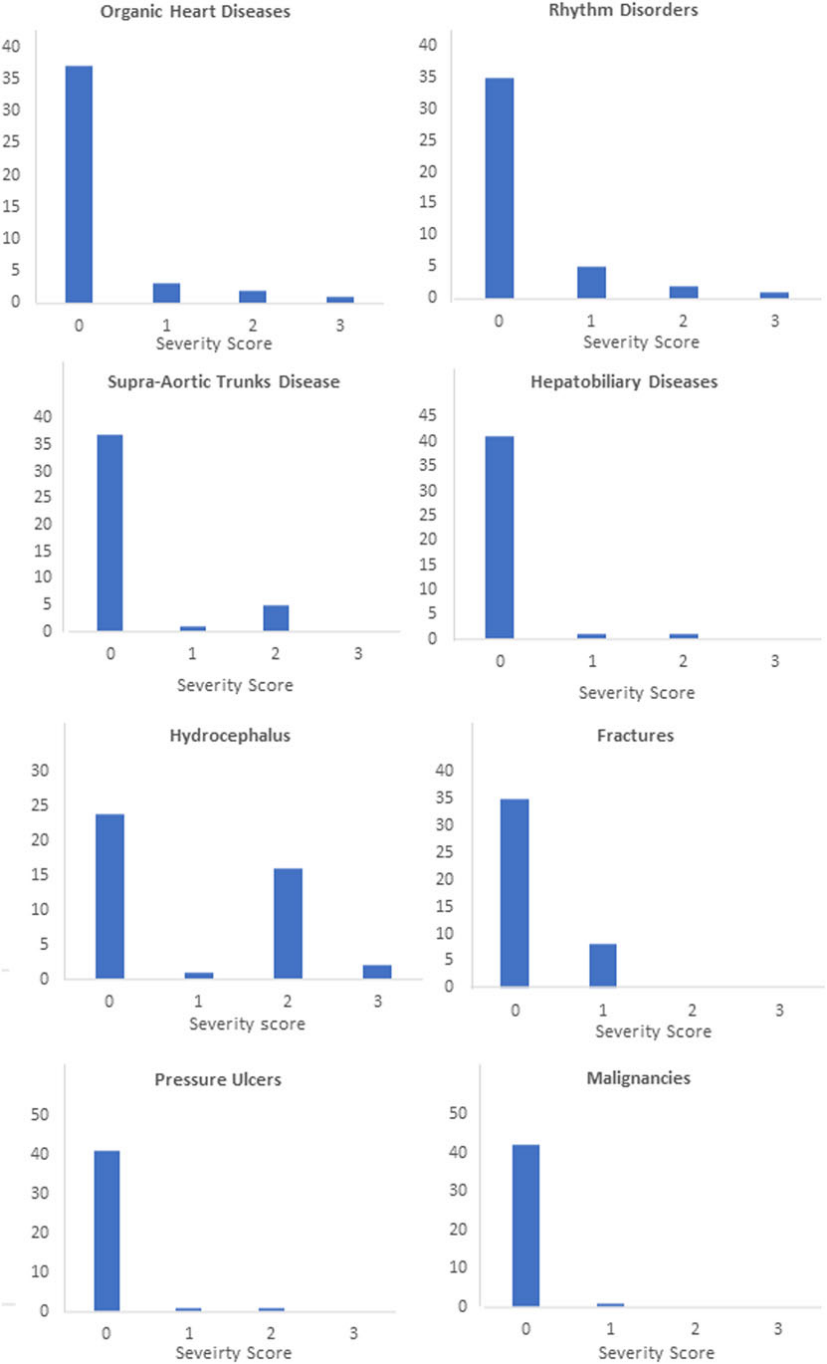
Severity distribution of comorbidities in the included patients.

The frequency and severity distribution of comorbidities are shown in Figures 1 and 2. Forty-two (98%) patients showed at least one comorbidity. No comorbidities were identified in one patient only. Peripheral artery diseases and peripheral vein diseases were not observed. Presence of life-support device (*n* = 39, 92.9%, 95% confidence interval [CI]: 2.19–2.80), anemia (*n* = 32; 76.2%, 95% CI: 0.73–1.16), arterial hypertension (*n* = 28, 66,7%, 95% CI: 1.07–1.73), hydrocephalus (*n* = 19, 45.3%, 95% CI: 0.59–1.26), and respiratory infections (*n* = 19; 45.2%, 95% CI: 0.33–0.75) were the comorbidities most frequently reported. The severity of the comorbidity was rated as mild in the whole sample (median total score 10, minimum 0–maximum 20): it was <10 (min-max, 0–10) in 24 patients (56%) and >10 (min-max, 12–20) in the remainder (44%).

The most frequent comorbidities by cause of injury were presence of life-support devices (*n* = 8; 100%), anemia (*n* = 7; 87%), dysautonomia (*n* = 6; 75%), and hydrocephalus (*n* = 5; 62%) in TBI; arterial hypertension (*n* = 5; 100%), presence of life-support devices (*n* = 5; 100%), rhythm disorders (*n* = 3; 60%), and organic heart diseases and supra-aortic trunks diseases (*n* = 2; 40%) in ischemic stroke; presence of life-support devices (*n* = 5; 83%), arterial hypertension (*n* = 4; 67%), and respiratory infections (*n* = 3; 50%) in ICH; and presence of life-support devices (*n* = 17; 85%), anemia (*n* = 15; 75%), arterial hypertension (*n* = 13; 65%), hydrocephalus (*n* = 11; 55%) in SAH; and respiratory infections, urinary tract infections, and supra-aortic trunk disease (*n* = 2; 100%) in perianeurysmal edema (PAE).

### Six-month outcome and impact of comorbidities

**FIG. 3.**
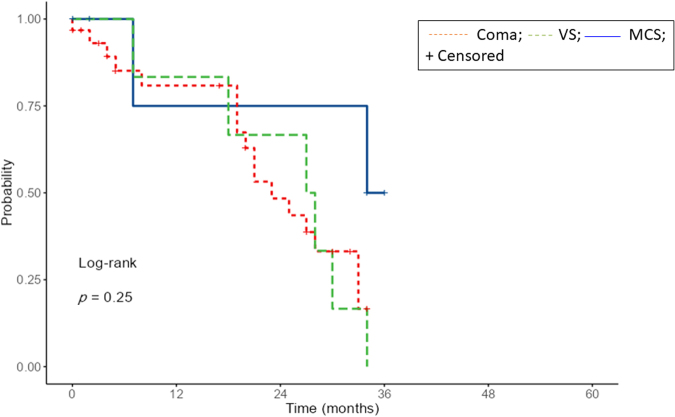
Kaplan–Meier's curves for survival according to different diagnoses.

At the 6-month follow-up, 19 patients had died (44%), 15 showed a full recovery of consciousness (35%), 7 were in a condition of emergence from MCS to full consciousness (16%), and 2 showed a persistent VS/UWS (5%). Supplementary Table S1 shows all the data of the patients included. A moderate negative correlation was found between age and initial CRS-R scores (Spearman's rho -0.397, *p* = 0.027) as well as between the presence of rhythm disorders and the initial GCS scores (Spearman's rho - 0.47; *p* = 0.038). A moderate positive correlation was found between age and the presence of diabetes (Spearman's rho 0.423; *p* = 0.005), rhythm disorders (Spearman's rho 0.330; *p* = 0.031) and organic hearth diseases (Spearman's rho 0.330; *p* = 0.031). As shown in Figure 3, the difference between the three Kaplan–Meier survival curves was not statistically significant (*p* = 0.25). The survival rate was 75.0% for patients in VS/UWS, 80.9% for patients in coma, and 83.3% for patients with MCS.

**FIG. 4.**
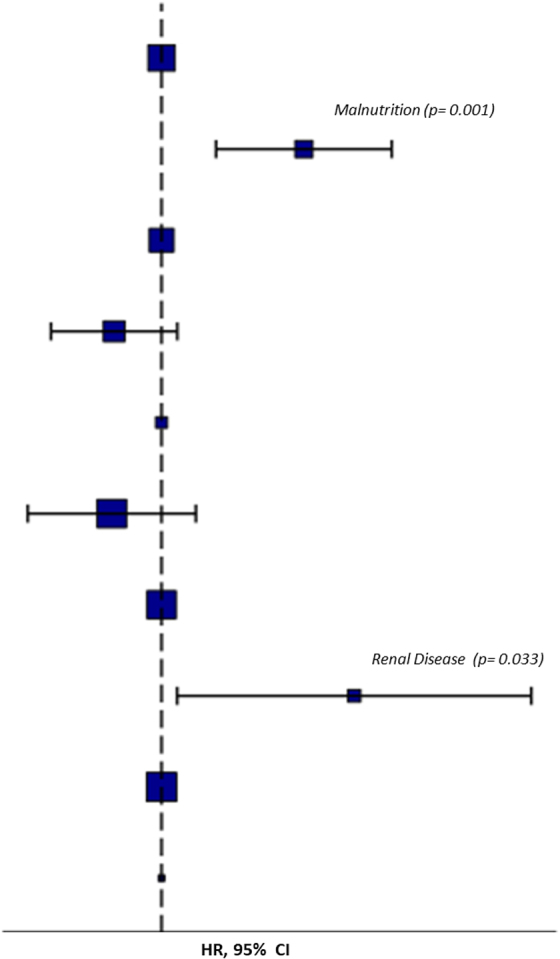
Predictors of missed recovery of consciousness at multivariable Cox analysis

The univariate Cox regression analysis showed that the presence of gastrointestinal disorders (HR = 27.46; *p* = 0.019), renal diseases (HR = 27.46; *p* = 0.019), and malnutrition (HR = 9.31; *p* = 0.002) were associated with a lower likelihood of full recovery of consciousness. On the other hand, the absence of dysautonomia (HR = 0.33; *p* = 0.059) and life support devices (HR = 0.27; *p* = 0.048) were positive predictors of full recovery of consciousness. Otherwise, the presence of supra-aortic trunk disease (HR = 3.24; *p* = 0.027) and of renal diseases (HR 6.05; *p* = 0.012) was associated with an increased risk of mortality, whereas a GCS score >5 (HR = 0.30; *p* = 0.026) was associated with a lower risk of mortality. At the multivariable Cox regression, the presence of renal disease (HR = 37.37; *p* = 0.033) and malnutrition (HR = 14.52; *p* = 0.001) were predictors of missed recovery of full consciousness (Fig. 4).

## Discussion

Our findings suggest that patients with DoC have multiple medical comorbidities that need to be closely monitored throughout the follow-up period. Previous evidence has suggested that some comorbidities may predict outcomes: in particular, the presence of ischemic or organic heart disease has been recognized as a predictor of death, whereas respiratory disease and arrhythmias have been reported as negative predictors of full recovery of consciousness and functional improvement.^6^ The presence of multiple comorbidities was confirmed in this study. The main outcomes were mainly predicted by the severity of brain damage, as expressed by initial GCS scores, but also by the presence of some clinical comorbidities. Specifically, renal disease and malnutrition, whenever diagnosed in patients, have been reported to decrease the chance for consciousness recovery.

Previous evidence came from studies conducted in rehabilitation care units, where patients had already survived the acute phase of severe acquired brain injury. In contrast, the present data were collected at an earlier stage of the disease, in an acute setting when patients were still in ICUs. It is likely that, in the acute phase, the severity of brain injury, as expressed by the initial GCS score, may be considered the main reliable predictor of outcomes and that comorbidities have a greater burden and influence in the long-term. Nevertheless, some comorbidities such as malnutrition and renal disease may play a pre-eminent role in the acute phase when patients are at a greater risk of being undernourished and dehydrated. Indeed, the mechanisms of recovery from DoC are different in the acute and subacute-to-chronic stages, and comorbidities may play a crucial role depending on the timing of assessment.

In this regard, the availability of CoCos, a scale specifically developed and validated for these patients, may help to increase the accuracy of prognosis in the various stages of the disease. As previously reported, DoC can be considered along a clinical continuum, and the principles and confounding factors in assessment, prognosis, and treatment change over time.^15,16^ The acute period of DoC lies with the first 28 days after injury, whereas the later period represents the subacute and chronic phases.^17^ In the acute phase of DoC, patients move from the site of injury to the emergency department and ICU. At this time, the severity of the brain injury has a great impact on survival and early recovery. Thereafter, patients move to rehabilitation hospitals, chronic care facilities, and sometimes home. During this period, patients may experience additional comorbidities, often resulting from prolonged immobility, which can negatively interfere with consciousness and functional recovery.

In our sample, the frequency and severity distribution of comorbidities across different patterns of brain injury reflects what was expected: it is interesting to highlight that almost all patients show at least one comorbidity and that the severity of each one was usually mild. This suggests that the presence of multiple comorbidities, rather than the severity of each single comorbidity, may play a role in determining the overall clinical complexity of patients. The burden of comorbidities, especially in the subacute and chronic stage, may limit access to rehabilitative sessions and influence the chances for recovery.^7,17,18^ Contrary to what observed in rehabilitative settings, some comorbidities such as peripheral vein diseases, were not observed and this difference is not surprising as peripheral vein disease is strongly associated with prolonged lack of movement in bedridden patients. The high use of life-support devices as well as the high frequency of anemia are consistent with the clinical profile of patients admitted in ICUs. The distribution of comorbidities across different patterns of brain injury may be explained by considering the pathophysiological mechanisms underlying the different diseases: dysautonomia and hydrocephalus are more likely to be recognized in TBI, whereas rhythm disorders, organic heart diseases, and supra-aortic trunk diseases are traditionally associated with ischemic stroke. Similarly, the presence of arterial hypertension is a well-known risk factor for ICH, and hydrocephalus is a common complication of SAH.

Strengths of our study include the rigorous selection of patients based on well-established criteria, the use of validated assessment tools for clinical evaluation, and longitudinal follow-up, which is not always feasible in these patients, who are usually transferred to places that are distant from the ICU. The choice to perform the study in an acute setting stemmed from the assumption that comorbidities may play a different role in acute and chronic stages; therefore, providing data in a setting different from those already explored in scientific literature contributes to new knowledge and awareness.

Limits of the study include the small sample of patients, which can be expanded in future. This reduces the generalizability of our findings to the whole population of severely injured patients in the acute setting. This is even more relevant with respect to the etiologies that were less represented in our sample as post-anoxic encephalopathy and metabolic encephalopathy.

To transfer our findings to a broader group of patients, the less represented subgroups have to be numerically increased. Moreover, when investigating the impact of comorbidities on outcomes we did not consider the total CoCos scores only but also the presence of single comorbidities at an individual level: in this analysis the smallness of the sample prompts caution in the interpretation of results that deserve further confirmation through large-sample prospective studies.

Moreover, not all the patients had an in-person evaluation at the 6-month follow-up: in a minority of cases, the outcome was identified through an interview with caregivers. However, we have specifically chosen clearly identifiable outcomes to minimize errors and confounding factors.

With these limits, our results confirm the clinical complexity of patients with DoC, both in the acute and in the subacute stages. The clinical scenario can progressively change as patients move from acute to subacute and chronic phases, with some comorbidities being resolved or replaced by others. Accurate prognostication of DoC requires a multi-faceted assessment, with particular attention being paid to primary brain injury, concomitant diseases, and previous and new-onset comorbidities that might have prognostic relevance.^19^ Prediction of outcomes is based on clinical assessment, brain imaging, electrophysiological studies, and analysis of chemical biomarkers.^15^ It is well known that mechanisms of recovery vary among disease etiologies, which makes post-anoxic encephalopathy the disease with the poorest chances for recovery contrary to TBI, which is characterized by the best opportunities for improvement.^20^ In fact, current guidelines usually indicate a poor prognosis for recovery after cardiac arrest, with only one third of patients regaining consciousness and with chances of recovering progressively decreasing over time. This led to establishing a deadline for improving prognosis of about 3 months after cardiac arrest and of about 12 months after TBI.^17^ However, etiologies alone do not predict final outcomes, as other clinical elements interfere with prognostic expectations. Therefore, unexpected recoveries may occur even in patients whose chances for recovery are exceedingly small.^20–22^ These prompt reflections on the need to avoid falsely pessimistic predictions and the self-fulfilling prophecy of a missed recovery.^23^ Considering the patient as a whole instead of focusing on brain injury alone is the best way to improve prognostication.

Research on comorbidities may improve our knowledge about the clinical profile of patients and its changes over time, with direct implications in clinical practice. The awareness of the clinical complexity of severely brain injured patients may improve care pathways and encourage an early and appropriate transition from acute care to rehabilitation settings.^7^ Moreover, evidence on the role of comorbidities on the clinical outcomes of patients with DoC needs to be integrated into a single conceptual framework, where all the useful elements for prognostication are considered together.

## Supplementary Material

Supplemental data

## Abbreviations Used

AE: anoxic encephalopathy
CI: confidence interval
CRS-R: Coma Recovery Scale – Revised
CoCos: Comorbidities Coma Scale
DoC: disorders of consciousness
GCS: Glasgow Coma Scale
HR: hazard ratio
ICH: intracerebral hemorrhage
ICU: intensive care unit
MCS: minimally conscious state
PAE: post-anoxic encephalopathy
SAH: subarachnoid hemorrhage
SD: standard deviation
TBI: traumatic brain injury
VS/UWS: vegetative state/unresponsive wakefulness syndrome
