# Supplementary material for: Assessment of Frequency and Predictive Value of Comorbidities in Patients With Disorders of Consciousness in the Acute Setting
Source: Neurotrauma Rep. 2024 Mar 14;5(1):267–76. doi: 10.1089/neur.2023.0120 (PMC10956526; doi:10.1089/neur.2023.0120)
Supplement: Supplemental data [file Suppl_TableS1.docx]

| N | SEX | Age | Primitive brain damage | Diagnosis | CoCos Total Score | Follow-up  Evaluation  Interview | Outcome |
| --- | --- | --- | --- | --- | --- | --- | --- |
| 1 | MALE | 68 | SAH | COMA | 21 | In person | Died |
| 2 | FEMALE | 57 | SAH | COMA | 15 | In person | Died |
| 3 | FEMALE | 84 | SAH | COMA | 9 | In person | Died |
| 4 | FEMALE | 58 | SAH | COMA | 13 | In person | Full recovery of consciusness |
| 5 | MALE | 77 | SAH | COMA | 9 | In person | Died |
| 6 | MALE | 17 | SAH | COMA | 9 | In person | Full recovery of consciousness |
| 7 | FEMALE | 34 | IS | COMA | 16 | In person | Full recovery of consciusness |
| 8 | MALE | 79 | TBI | MCS | 10 | In person | Died |
| 9 | FEMALE | 66 | SAH | COMA | 13 | In person | Full recovery of consciusness |
| 10 | MALE | 75 | SAH | COMA | 13 | In person | Full recovery of consciusness |
| 11 | MALE | 22 | TBI | COMA | 9 | In person | Full recovery of consciusness |
| 12 | MALE | 58 | SAH | COMA | 9 | In person | Full recovery of consciusness |
| 13 | MALE | 75 | TBI | VS/UWS | 14 | In person | Died |
| 14 | MALE | 36 | TBI | VS/UWS | 13 | In person | Died |
| 15 | FEMALE | 84 | TBI | COMA | 7 | In person | Died |
| 16 | MALE | 69 | SAH | VSUWS | 4 | In person | Full recovery of consciousness |
| 17 | MALE | 70 | SAH | VS/UWS | 10 | In person | Died |
| 18 | FEMALE | 84 | IS | COMA | 13 | In person | Died |
| 19 | MALE | 39 | SAH | MCS | 8 | In person | Full recovery of consciousness |
| 20 | MALE | 20 | TBI | COMA | 7 | In person | Unchanged |
| 21 | FEMALE | 51 | ICH | COMA | 0 | In person | Full recovery of consciousness |
| 22 | FEMALE | 62 | ICH | COMA | 9 | In person | Died |
| 23 | MALE | 43 | ICH | MCS | 6 | In person | Full recovery of consciousness |
| 24 | MALE | 43 | SAH | COMA | 7 | In person | Died |
| 25 | MALE | 50 | SAH | COMA | 7 | In person | Died |
| 26 | MALE | 31 | SAH | MCS | 4 | In person | Full recovery of consciousness |
| 27 | FEMALE | 74 | TBI | COMA | 8 | In person | Emergence from MCS |
| 28 | FEMALE | 69 | SAH | MCS | 13 | In person | Full recovery of consciusness |
| 29 | FEMALE | 59 | TBI | COMA | 10 | In person | Unchanged |
| 30 | FEMALE | 80 | SAH | COMA | 12 | In person | Died |
| 31 | FEMALE | 85 | SAH | VS/UWS | 10 | In person | Died |
| 32 | FEMALE | 52 | ME | MCS | 12 | In person | Full recovery of consciousness |
| 33 | FEMALE | 63 | ICH | COMA | 16 | In person | Emergence from MCS |
| 34 | FEMALE | 23 | PAE | VS/UWS | 16 | In person | Emergence from MCS |
| 35 | FEMALE | 62 | PAE | COMA | 20 | In person | Emergence from MCS |
| 36 | FEMALE | 97 | IS | COMA | 12 | In person | Emergence from MCS |
| 37 | MALE | 86 | IS | COMA | 18 | In person | Died |
| 38 | FEMALE | 101 | IS | COMA | 4 | In person | Died |
| 39 | MALE | 75 | ICH | COMA | 14 | In person | Died |
| 40 | FEMALE | 65 | ICH | COMA | 10 | Caregivers | Emergence from MCS |
| 41 | FEMALE | 65 | SAH | COMA | 10 | Caregivers | Emergence from MCS |
| 42 | MALE | 41 | SAH | COMA | 5 | Caregivers | Full recovery of consciusness |
| 43 | MALE | 48 | IS | COMA | 6 | Caregivers | Died |

SAH: subarachnoid hemorrhage; TBI: traumatic brain injury; IS: ischemic stroke; ICH: intracerebral hemorrhage; ME: metabolic encephalopathy; PAE: post-anoxic encephalopathy.
